# Therapeutic efficacy of the humanized JAA-F11 anti-Thomsen-Friedenreich antibody constructs H2aL2a and H3L3 in human breast and lung cancer xenograft models

**DOI:** 10.18632/oncotarget.28282

**Published:** 2022-10-19

**Authors:** Diala Ghazal, Fatma Zalzala, John C. Fisk, Swetha Tati, Loukia G. Karacosta, Susan Morey, James R. Olson, Sally Quataert, Grace K. Dy, Kate Rittenhouse-Olson

**Affiliations:** ^1^For-Robin, Inc, Williamsville, NY 14221, USA; ^2^Department of Biotechnical and Clinical Laboratory Sciences, University at Buffalo, Buffalo, NY 14214, USA; ^3^Department of Pharmacology and Toxicology, University at Buffalo, Buffalo, NY 14203, USA; ^4^Department of Microbiology and Immunology, David H. Smith Center for Vaccine Biology and Immunology, University of Rochester, Rochester, NY 14642, USA; ^5^Department of Medicine, Roswell Park Comprehensive Cancer Center Buffalo, NY 14203, USA

**Keywords:** hJAA-F11, TF-Ag, Thomsen-Friedenreich antigen, tumor immunotherapy, translational oncology

## Abstract

The Thomsen-Friedenreich antigen (TF-Ag-α) is found on ~85% of human carcinomas but is cryptic on normal tissue. The humanized highly specific hJAA-F11-H2aL2a and -H3L3 antibodies target TF-Ag-α without binding to TF-Ag-beta (found on surface glycolipids of some normal cells). The relative affinity of H3L3 is 17 times that of H2aL2a, which would seem to favor superior efficacy, however, increased affinity can result in less tumor penetration. To assess the potential therapeutic efficacy of these antibodies, four human cancer- mouse xenograft models were treated with H2aL2a and H3L3.

The tumor xenograft models used were human non-small cell lung cancer, H520, and small cell lung cancer, HTB171 in nude mice and human triple negative breast cancer, MDA-MB-231 and HCC1806 in SCID mice. H2aL2a significantly decreased tumor growth in both breast and both lung cancer models. H2aL2a showed statistically equal or better efficacy than H3L3 and has superior production capabilities. These results suggest that H2aL2a may be superior as a naked antibody, as an antibody drug conjugate or as a radiolabeled antibody, however the higher affinity of H3L3 may lead to better efficacy in bi-specific therapies in which the binding is decreased due to the presence of only one TF-Ag-α binding site.

## INTRODUCTION

The disaccharide D-galactose-beta-(1-3)-N-acetyl galactosamine-alpha (Gal-β-(1-3)-GalNAc-α), known as Thomsen-Friedenreich antigen (TF-Ag-α) or Core 1, is found on approximately 85% of human carcinomas but not on normal tissues. This makes TF-Ag-α a promising target for cancer therapeutics. Alterations in glycosylation in malignant cells lead to the elevated surface expression of TF-Ag-α on cancer cells, while TF-Ag-α is naturally cryptic on normal tissues due to glycan chain extensions. [1–3] However, previous attempts at therapy utilizing this target have been limited due to a lack of chemical and biological specificity of earlier antibodies developed to this target. Our patented humanized IgG_1_ antibody constructs H2aL2a and H3L3 (hJAA-F11 H2aL2a and hJAA-F11 H3L3) are the only humanized antibodies that are highly specific for true tumor associated TF-Ag alpha [4, 5]. Importantly, these antibodies do not bind to TF-Ag beta which is primarily linked to glycolipids on the surface of normal cells such as the central nervous system GM1 ganglioside, the asialo-GM1 of NK and kidney cells, the GD1 of glycolipids and the asialo- GM1 of peripheral nerve tissue. Thus, hJAA-F11 antibodies, the humanized versions of the murine monoclonal IgG_3_ antibody mJAA-F11 [4–12], hold promise towards targeting TF-Ag-α expressing cancers for therapeutic and diagnostic applications.

During humanization of the mouse JAA-F11, 4 different constructs of the heavy chain and 4 different constructs of the light chain were made. The various combinations were tested and H2aL2a and H3L3 were selected as the most reactive to TF-Agα in an ELISA with TF-Ag-α-BSA as the target antigen [4].

Both H2aL2a and H3L3 showed antibody-dependent cellular cytotoxicity (ADCC) activity *in vitro*, with H3L3 being superior [4]. The amino acid differences in the V framework region results in the relative affinity of H2aL2a to be 1.9 times the mouse JAA-F11 (mJAA-F11), and the relative affinity of H3L3 to be 33 times that of the mJAA-F11 [4]. The higher affinity and *in vitro* ADCC activity of H3L3 would seem to be predictive of improved therapeutic effect for this construct. However, previous work with other cancer therapeutic antibodies [13–15], has shown that beyond a certain affinity threshold, increased affinity can result in less penetration in the tumor due to the binding-site barrier phenomenon, causing less efficacy. This decrease in efficacy seen with higher affinity has been suggested to be a deficiency that would globally affect all tumor types [13–15]. To test for the potential therapeutic efficacy and to determine how the difference in affinity affects the efficacy of H2aL2a and H3L3, four clinically relevant human cancer mouse xenograft models were treated with each antibody construct.

*In vivo* efficacy of the H2aL2a construct has been previously shown in the human triple negative breast cancer (TNBC) MDA-MB-231 xenograft in Severe Combined Immunodeficient (SCID) mice [4]. In this report, the efficacy of both H2aL2a and H3L3 are compared in lung and breast cancer human tumor xenograft models in immunocompromised mice. The tumor models utilized, both non-small cell squamous cell carcinoma and small cell lung cancer and triple negative breast cancer are tumor types in which there is an unmet clinical need for targeted therapy. In immunohistochemical analysis of human tumors as shown in our previous work [5], these selected tumor types had high percentage of TF-Ag expression (specific reactivity with hJAA-F11 H2aL2a). 52 Individual cases of non-small cell squamous carcinomas were tested with hJAA-F11 H2aL2a and 96% were positive, 38 individual cases of small cell carcinomas were tested and 97% were positive. 126 individual cases of triple negative breast tumors were tested and 94% were positive, Accordingly, the human non-small cell lung cancer (NSCLC, (squamous cell), H520) and small cell lung cancer (SCLC, HTB171) xenografts in nude mice and the TNBC xenografts MDA-MB-231 and HCC1806 in SCID mice were used in this study.

## RESULTS

### Antibody production

Transient expression of H2al2a and H3L3 was initially performed with the pFUSE-ChIg-HG1/pFUSE2-CLIG-hK vector (InVivogen Grand Island, NY USA) using the ExpiCHO-S transient cell line, which yielded 15 mg/L for H2aL2a and 0.4 mg/L for H3L3. Subsequently the expression was performed using the pcDNA3.4 (Thermo Fisher Scientific, Grand Island, NY, USA) vector with Thermo Fisher’s codon optimization Geneart service, which yielded 1160 mg/L for H2aL2a and 40 mg/L for H3L3. This was a satisfactory yield for H2aL2a but low for H3L3. In attempt to improve yield of H3L3, Aragen (Aragen Bioscience Morgan Hill, CA, USA) vectors and codon optimization were used for H3L3, and the yield was still low at 19 mg/L. Genescript (Piscataway, NJ, USA) vectors were also used with H3L3 with CHO cells in transient expression and the yield was again low at 1.2 mg/L. Thus, satisfactory yield was found for H2aL2a but not for H3L3.

### Xenograft tumor cell characteristics

#### TF-Ag-α target number on tumor cell lines

**Figure 1:**
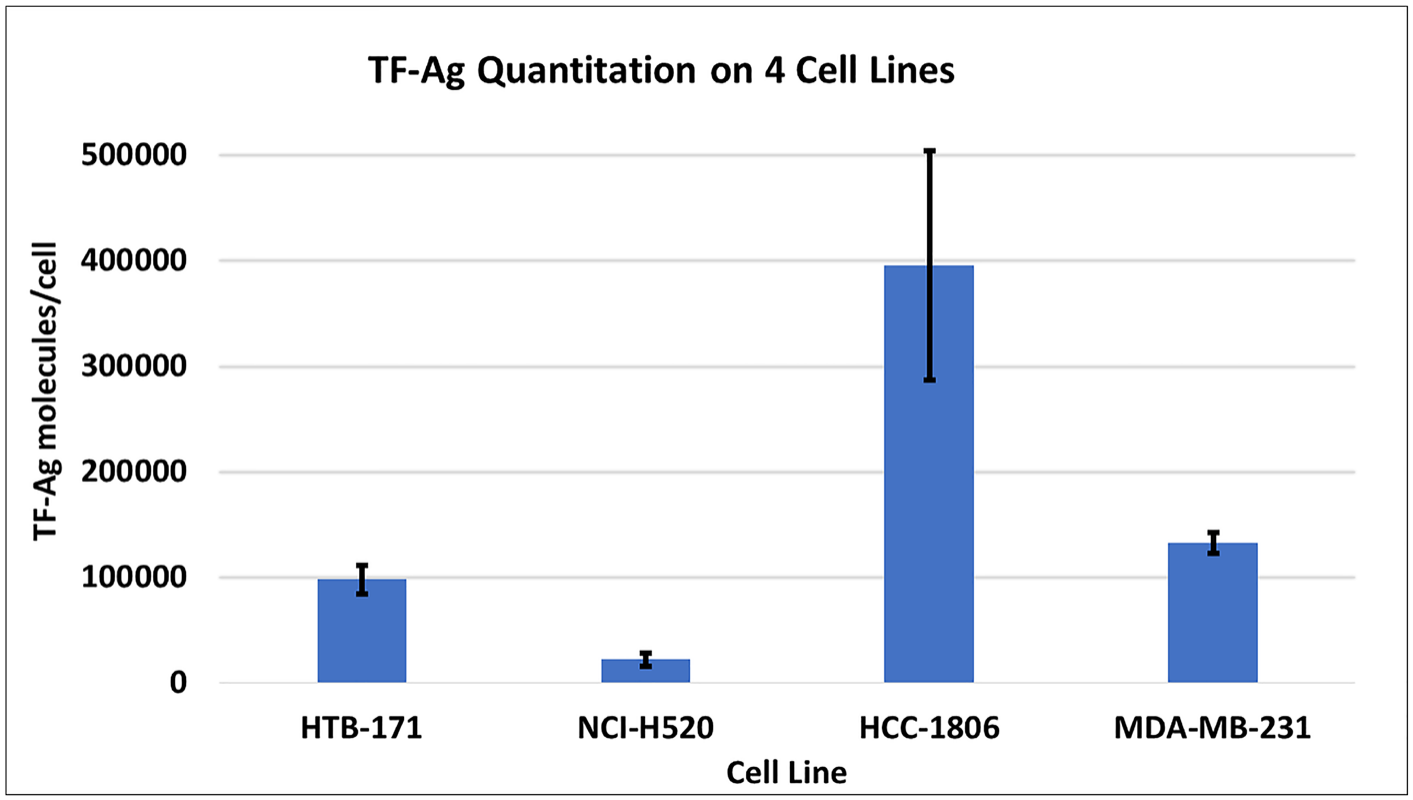
Quantitation of TF-Ag molecules per cell using JAA-F11 in the Bangs method. Each cell line was harvested using Cell Stripper rather than trypsin to minimize loss of glycoproteins. The number of individual experiments per cell line MDA-MB-231 = 11, HCC-1806 = 7, HTB-171 = 4, NCI-520 = 4, Values represent the mean +/−SEM.

To confirm and quantify TF-Agα expression (target molecules per cell) on the cancer cell lines employed in this study, the Bangs method was used (Figure 1). The relative number of TF-Ag-α targets per cell on the cell lines used in the *in vivo* efficacy studies herein, are as follows: HCC-1806 (395,547) > MDA-MB 231 (132,791) > HTB-171 (98,251) > H-520 (22,166).

### *In vivo* tumor doubling time of human tumor models

The method of Gordon et al., [16] was used to determine the *in vivo* doubling time of the cell lines utilized in these experiments. The tumor doubling time in the untreated mice are shown in Table 1. MDA-MB-231(5.64 days) and HCC-1806 (5.08 days) have nearly equal doubling times, whereas H-520 (3.14 days). and HTB-171 (2.85 days) have a faster doubling time.

**Table 1:** Summary of cell line and antibody *in vivo* efficacy comparison in xenograft tumor models

Cell line	Mouse model	Ranked effect of antibodies	TF-Ag expression	Doubling time (Days)	Antibody with best *in vivo* efficacy
HTB171	Nude	1	++	2.85	H2aL2a = H3L3
H520	Nude	2	+	3.14	H2aL2a
HCC1806	SCID	3	+++	5.08	H2aL2a = H3L3
MDA-MB231	SCID	4	++	5.64	H2aL2a

Ranked efficacy in each model, TF-Ag expression, *In vivo* Tumor Doubling Time (TDT) and hJAA-F11 construct with the best efficacy in reducing tumor volume in the 4 mouse xenograft models. The interval used to determine tumor doubling time in control mice was the difference between the first day of volume measurement and last day of volume measurement before the first mouse was sacrificed.

### Efficacy of hJAA-F11- H2aL2a and - H3L3 antibodies in human NSCLC and SCLC xenograft models

**Figure 2:**
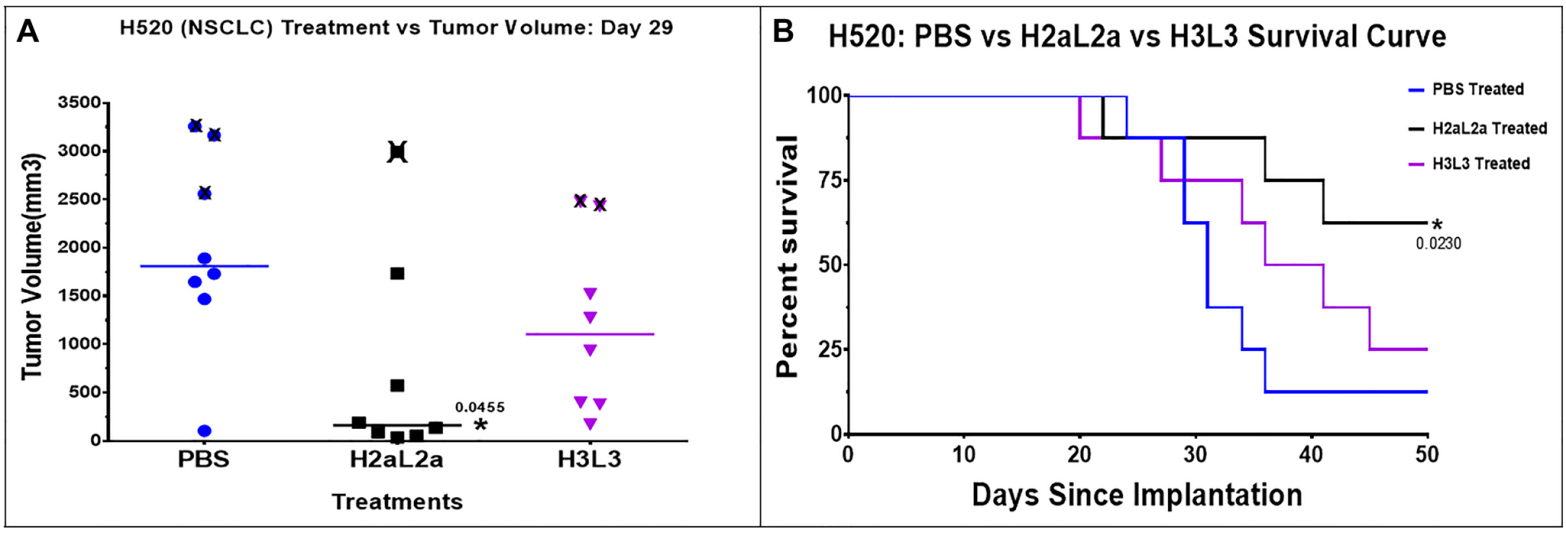
Efficacy of hJAA-F11- H2aL2a and - H3L3 in the H520 human squamous cell non-small cell lung cancer (NSCLC) nude mouse xenograft model. (**A**) Scatter dot plot of tumor volume shown at day 29. “X” represents animals sacrificed after this time point due to large tumor size (≥2000 mm^3^). H2aL2a significantly reduced tumor volume relative to the PBS control group (*p* = 0.0455; one-way ANOVA with Dunnett’s test). The tumor volumes were compared at this day and not later due to the sacrifice of some of the animals due to large tumor size. (**B**) Kaplan-Meier survival plot shows that H2aL2a treatment significantly prolonged survival in this aggressive tumor model. Mantel – Cox Chi Square was used to determine statistical significance between the survival of the experimental and the control groups.

The efficacy of H2aL2a and H3L3 in the NSCLC H-520 xenograft model is shown in Figure 2. H2aL2a treated animals showed decreased tumor size (Figure 2A) and significantly improved survival (Figure 2B) when compared to controls, while H3L3 treatment did not statistically improve survival when compared to the PBS control group (Figure 2B). H-520 is a rapidly growing tumor with a large variation in tumor size, so this experiment was repeated 3 times. Statistically significant effect versus the PBS control was shown for H2aL2a in 2 of 3 experiments, whereas H3L3 treatment did not show statistically significant effects in any of the experiments (See Supplementary Data 1 for raw data for this experiment).

**Figure 3:**
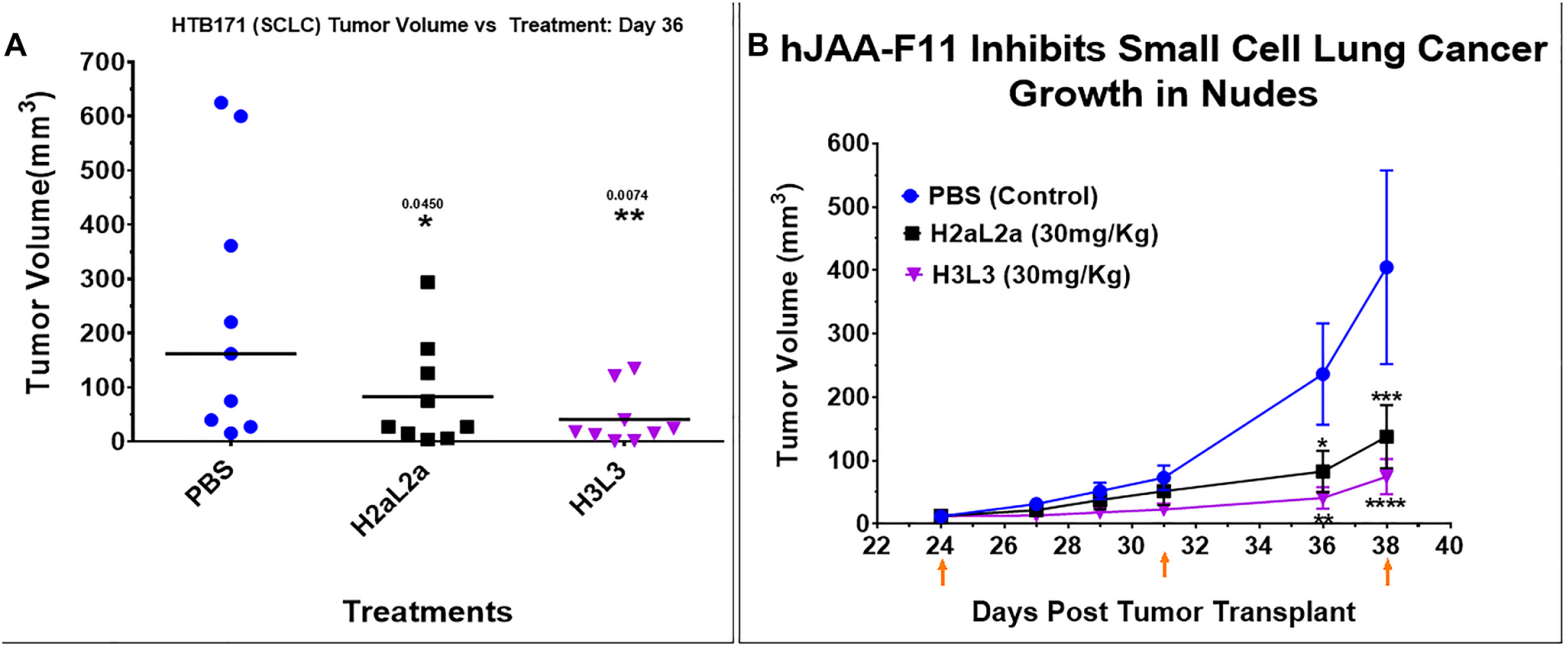
Efficacy of hJAA-F11- H2aL2a and - H3L3 in HTB171 human small cell lung cancer (SCLC) in nude mouse xenograft model. (**A**) Scatter dot plot of tumor volume shown at day 36. Treatments with H2aL2a and H3L3 significantly reduced tumor volume relative to the PBS control group (one-way ANOVA with Dunnett’s multiple comparisons). (**B**) Line graph of tumor volume on days 24–38 after tumor cell inoculation. The tumor volume (mean ± SEM) of the H2aL2a and H3L3 groups were significantly different than the PBS control mice on day 36 and 38 (^*^ *p* < 0.05, ^**^ *p* < 0.01, ^****^ *p* < 0.0001; two-way ANOVA with Dunnett’s test). Arrows indicate treatment days.

The efficacy of hJAA-F11- H2aL2a and - H3L3 in the nude mouse xenograft model with HTB-171 human SCLC is shown in Figure 3. Figure 3A shows a scattergram with the tumor size of each mouse shown at day 36. Figure 3B shows a time course of tumor volume with days post tumor treatment for each of the treatment groups. Comparison of mean tumor volume between the groups indicated that H2aL2a and H3L3 had significantly (*p* = 0.0001) lower mean tumor volumes than the PBS control group, while there were no significant differences in the tumor volumes between the H2aL2a and H3L3 groups (See Supplementary Data 1 for raw data for this experiment).

### Efficacy of hJAA-F11- H2aL2a and - H3L3 antibodies in human TNBC xenograft models

**Figure 4:**
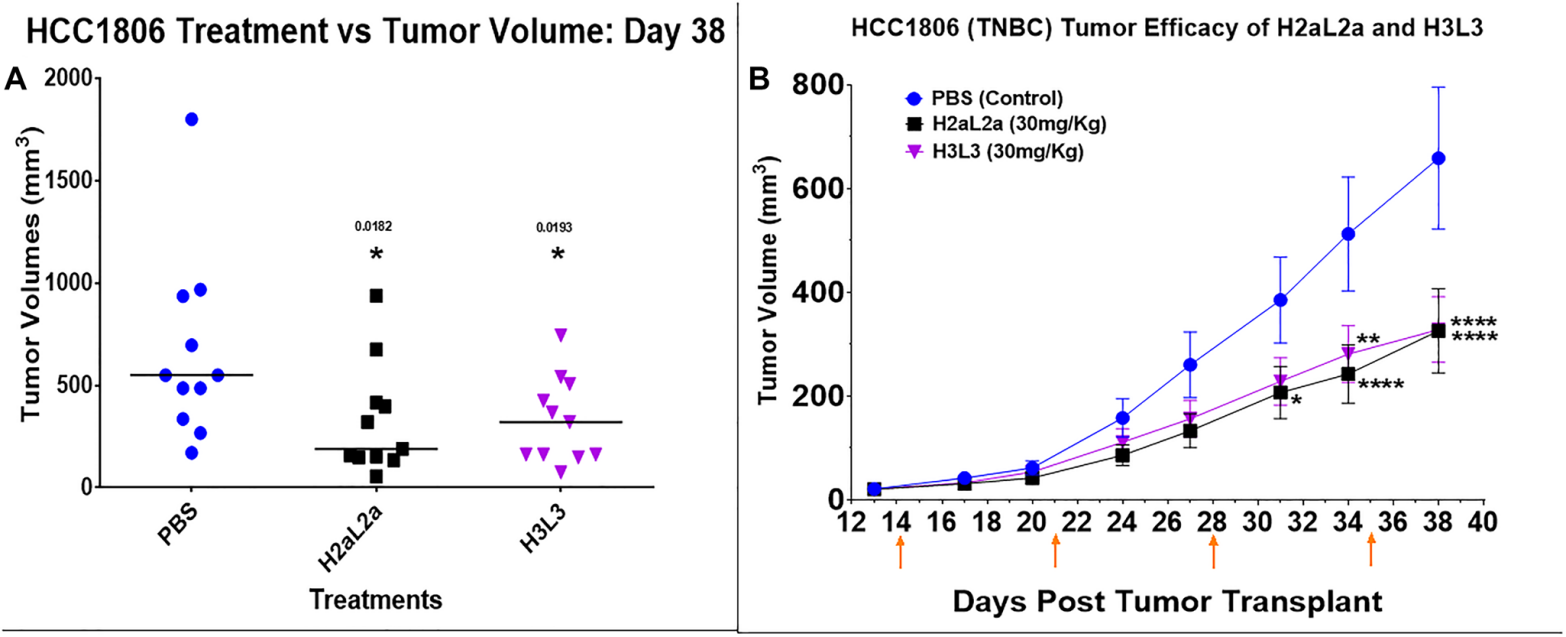
Efficacy of hJAA-F11- H2aL2a and - H3L3 in the HCC-1806 human triple negative breast cancer SCID mouse xenograft model. (**A**) Tumor volume scatter dot plot at 38 days following tumor cell inoculation. There were 11 mice per group. Both constructs produced similar significant decreases in tumor growth compared to the PBS control mice (*p* < 0.05, one-way ANOVA with Dunnett’s test). (**B**) The tumor volume (mean ± SEM) of both the H2aL2a and H3L3 groups were significantly different than the PBS control mice on the days indicated by asterisks (^*^ *p* < 0.05, ^**^ *p* < 0.005, ^****^ *p* < 0. 0001; two-way ANOVA with Dunnett’s test)). The experiment was terminated on 38 days due to ulcerations at the tumor site in several animals. On day 14, the first day of treatment, tumor size averaged 21 mm^3^. Arrows indicate treatment days.

In the experiments in HCC-1806 human triple negative breast cancer xenografts, both constructs produced similar significant decreases in tumor growth compared to the PBS control mice (Figure 4A, 4B). Individual tumor volumes are shown in the scattergram 4A, and a time course is shown in 4B. The experiment terminated on 38 days due to ulcerations at the tumor site in several animals (See Supplementary Data 1 for raw data for this experiment).

**Figure 5:**
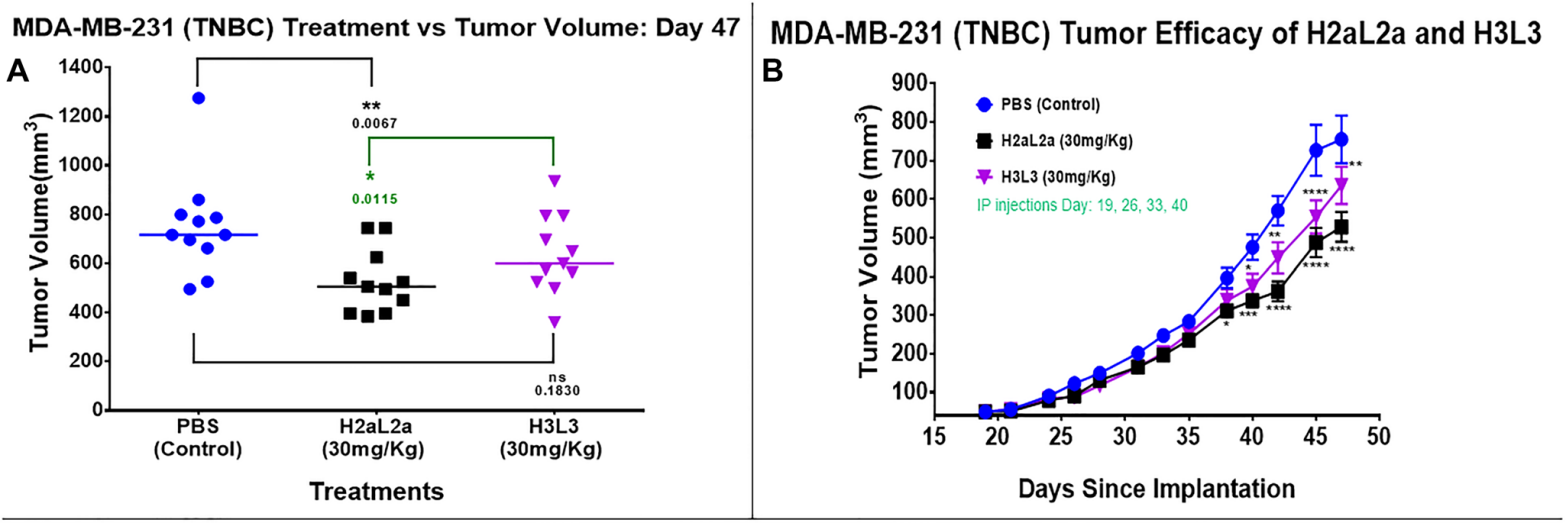
Efficacy of hJAA-F11- H2aL2a and - H3L3 in the MDA-MB-231 human triple negative breast cancer SCID mouse xenograft model. (**A**) Tumor volume scatter dot plot at 47 days following tumor inoculation. Mean tumor volume in the group treated with H2aL2a was significantly smaller than both the PBS control and H3L3 groups (one-way ANOVA with Dunnett’s test; *n* = 11 mice/group). (**B**) Line graph of tumor volume on days 19–47 after tumor cell inoculation. The tumor volume (mean ± SEM) of both the H2aL2a and H3L3 groups were significantly different than the PBS control mice on the days indicated by asterisks (^*^ *p* < 0.05, ^**^ *p* < 0.01, ^****^ *p* < 0.0001; two-way ANOVA with Dunnett’s test).

In our previous paper [4], H2aL2a had successfully decreased the growth rate of MDA-MB-231 cells in SCID mice, however the treatment in the previous work began on day 7 when the tumors were first palpable. The present study used a more clinically relevant model, with larger MDA-MB-231 tumors, averaging 49 mm^3^ at the time of initiating antibody treatment, in addition, H3L3 treatments were performed to compare the efficacy of the 2 different hJAA-F11 constructs (Figure 5). H2aL2a caused a statistically significant decrease in tumor size compared to PBS control mice at Day 47 (*p* = 0.0067) but H3L3 did not have a significant effect when compared to PBS on this day (*p* = 0.1830) (Figure 5A). In addition, the mean tumor volume of the H2aL2a group was significantly smaller compared to the H3L3 group on day 47 (*p* = 0.0115). In the time course experiment, both H2aL2a and H3L3 are shown to cause a significant decrease in the tumor volume relative to the PBS group after day 38 for the H2aL2a group and after day 40 for the H3L3 group (See Supplementary Data 1 for raw data for this experiment).

## DISCUSSION

The potential therapeutic efficacy of the humanized antibodies to TF-Ag-α, hJAA-F11-H2aL2a and hJAA-F11-H3L3, was assessed in human breast and lung cancer xenograft models. Our previous studies showed that the relative affinities of humanized constructs H2aL2a and H3L3 for TF-Ag-α were 1.9 and 33.3 times greater than that of the mouse JAA-F11 antibody, respectively [4]. Although both H2aL2a and H3L3 show ADCC activity *in vitro*, H3L3 is superior for ADCC *in vitro* [4]. Since higher *in vitro* affinity and higher ADCC activity does not necessarily correlate with optimum *in vivo* activity [13–15], the *in vivo* efficacy of the H2aL2a and H3L3 constructs were compared in mouse xenograft cancer models generated from 4 human tumor cell lines. H2aL2a effectively decreased tumor growth rate in the 2 breast cancer and 2 lung cancer models tested. In addition, production yields of the antibodies were compared and the yield of H2aL2a was 29 times that of the best yield of H3L3, indicating an advantage for the clinical development of H2aL2a.

In the H-520 human squamous cell NSCLC xenograft model in nude mice, H2aL2a significantly decreased tumor growth and improved survival when compared to the PBS control group. H3L3 treatment reduced tumor size and improved survival but neither change with H3L3 was significant when compared to the PBS control (Figure 2A, 2B). In contrast, the efficacy of hJAA-F11 - H3L3 and H2aL2a in the nude mouse xenograft model with HTB-171 human SCLC were both significant compared to the PBS control. Comparison of mean tumor volume between the groups indicated that H3L3 and H2aL2a both had significantly (*p* = 0.0001) lower mean tumor volume than the PBS control group, while there were no significant differences in the tumor volumes between the H2aL2a and H3L3 groups (Figure 3A, 3B).

In the HCC-1806 TNBC xenograft model, both H2aL2a and H3L3 significantly decreased tumor size when compared to the PBS control group (Figure 4). There was no difference in the efficacy of these constructs, with H2aL2a and H3L3 treatment producing a similar reduction in tumor size.

In the MDA-MB-231 TNBC xenograft model in SCID mice, the efficacies of H3L3 and H2aL2a in reducing tumor volume were determined by comparing tumor volumes to those of the control group receiving PBS (Figure 5). A significant reduction in tumor volume was observed from day 40 until the end of the experiment on day 47 for both antibody constructs relative to the control PBS group. On the last day of the study, day 47, the H2aL2a treated animals had a statistically significantly smaller tumor size than the H3L3 treated animals. The cumulative results suggest that H2aL2a, although of a lower affinity than H3L3, may be more effective at penetrating the tumors and thereby reducing tumor volume.

The response to H2aL2a therapy was greater in the nude mouse lung cancer models (H520 and HTB-171) than it was in the triple negative breast cancer SCID mouse models (HCC-1806, MDA-MB-231). While this effect may be due to tumor type, the effect may also be due to the host mouse strain. The SCID mouse model has been found to permit more types of human tumor growth than nude mice and in our hands SCID mice were a better host for the MDA-MB-231 tumor growth [17]. While both nude mice and SCID mice are immunodeficient, with nude mice lacking T cells and SCID mice lacking both T and B cells, they both have NK cells necessary for ADCC [18]. Similar to what was observed in our models, in a study by Barok et al, Trastuzumab (Herceptin) was found to have a greater effect on JIMT-1 breast cancer in nude mice than in SCID mice. They suggested that this was related to decreased immunity in the SCID mice in comparison to the nude mice [19].

TF-Ag-α target number was also assessed for each of the 4 human tumor cell lines. The relative number of TF-Ag targets was highest in HCC1806 cells followed by MDA-MB-231 > HTB-171 > H-520. Comparing efficacy of the antibodies to reduce tumor growth in the four *in vivo* models would suggest that the relative TF-Ag expression was not directly related to *in vivo* efficacy, since H2aL2a was efficacious in all models, and H3L3 had statistically significant effects in HCC1806, HTB171 and MDA-MB-231 and the effect of H3L3 was less than H2aL2a for MDA-MB-231 on the last day of the experiment. However, TF-Ag expression is related to efficacy when the experiments in the 2 mouse strains are compared separately. In nude mice, while HTB-171 has higher TF-Ag expression than H520, H2aL2a was similarly very effective in both, while H3L3 was more effective in reducing tumor growth in the higher expressing HTB-171 model. In SCID mice, both antibodies appear more efficacious in the higher TF-Ag expressing HCC1806 model than the MDA-MB-231 tumor model. Viewing the data from the different mouse strains separately is supported by data from others which show a difference in response of the different types of immunodeficient mice [19]. Thus, if the four tumor models are stratified by mouse strain, TF-Ag expression is related to efficacy.

*In vivo* tumor growth rate was compared in the PBS groups of the HTB-171, H-520, HCC-1806 and MDA-MB-231 models, to determine if growth rate influenced which antibody had superior efficacy. Since MDA-MB-231 and HCC-1806, both in SCID mice, have nearly equal doubling times, and HTB-171 and H-520 both in nude mice, have a faster doubling time it does not appear that tumor growth rate was related to whether H3L3 was as efficacious as H2aL2a. Table 1 summarizes the *in vivo* tumor doubling time, the relative TF-Ag expression for each tumor cell type and the resulting efficacy of H2aL2a and H3L3 in the 4 human tumor xenograft models in mice. In each mouse model, SCID or nude, treatment of the tumor with the higher TF-Ag expression showed higher efficacy. In each mouse model the efficacy of H2aL2a was either equal to or better than H3L3. We hypothesize that the lower affinity antibody (hJAA-F11 H2aL2a) may be the optimum way to treat the tumor, as better penetration may occur. For bi-specific molecules, the higher affinity antibody (hJAA-F11 H3L3) may be a better choice since the effective binding would be decreased from two H3L3 paratope-TF-Agα epitope interactions to one for the bi-specific.

In conclusion, both H2aL2a and H3L3 humanized antibodies to TF-Ag-α show efficacy in *in vivo* xenograft models of human tumors in SCID and nude mice and thus hold promise as therapeutics for breast and lung cancer. H2aL2a significantly decreased tumor growth rate in both breast cancer and both lung cancer models tested. H2aL2a is equal to or better than H3L3 in four of the four models and H2aL2a cell lines have far superior antibody production capabilities under the conditions tested.

The effect of human anti-tumor immunoglobulin in mouse models has been found to be less than that of the similar mouse anti-tumor immunoglobulin [20–22]. This lesser effect would be expected in our mouse models both because of the different reactivity of human and mouse Fc regions with mouse white blood cells, and because of the immunodeficient state of both the SCID and nude mice utilized. The better response in nude mice compared to SCID mice, is expected to indicate even a greater response in humans in the clinic due to the improved reaction of human Fc receptors with human antibody, and the improved immune reactivity in immunocompetent humans compared to immunodeficient mice. Thus, we expect a greater response in the clinic than was seen in the mouse models.

Together, the results suggest that H2aL2a may be superior when utilized as a naked antibody, as an antibody drug conjugate or as a radiolabeled antibody, whereas the higher affinity of H3L3 may lead to higher efficacy as one partner in bi-specific therapies to target TF-Ag-α on tumors.

## MATERIAL AND METHODS

### Antibody production

#### Production of hJAA-F11 H2aL2a and H3L3

Transient expression of H2aL2a and H3L3 was performed with multiple production vector constructs to maximize antibody production. Initially, transient expression of H2aL2a and H3L3 was performed with the pFUSE-ChIg-HG1/pFUSE2-CLIG-hK vectors (Invivogen, San Diego, CA, USA) using the ExpiCHO-S transient cell line (Thermo Fisher Scientific, Grand Island, NY, USA) as well as vectors from Genescript (Piscataway, NJ, USA). In an independent attempt to improve yield, vectors and codon optimization were also attempted by Aragen (Aragen Bioscience Morgan Hill, CA, USA). Due to low mg/L production, production was subsequently performed using the pcDNA3.4 (Invitrogen, Grand Island, NY, USA) vector with Invitrogen’s codon optimization Geneart service, all within the ExpiCHO expression system. In all production runs, H2aL2a consistently displayed higher production outputs than H3L3.

### Purification of antibody

Antibody was purified from production supernatants using MabSelect protein A column (Cytiva Marlborough, MA, USA) according to manufacturer recommendations. Prior to use in the mice, antibodies were dialyzed against 1× PBS and purity determined using SDS-PAGE electrophoresis and Coomassie staining analysis. Antibody purity used in *in vivo* studies was 95% or greater (See Supplementary Data 2 and 3 for the SDS-PAGE gels and analysis. Expicho h2al2a #2.3 vs. BSA 020618 lane report and expiCHOH3L3 #2 vs. BSA vs. FT 102617 Lane Report).

### Cell line characteristics

#### Bangs method for quantitation of TF-Ag target number on tumor cell lines

Single cell suspensions of *in vitro* propagated adherent cancer cell lines were prepared using Cell Stripper solution (Corning, Glendale, Arizona). After removal from the tissue culture flask, cells were kept at 4°C and washed twice with wash buffer (DPBS/2%BSA) before resuspending at about 1 × 10^7^ cells/mL in 0.4% rat IgG/PBS/2%BSA blocking buffer. The cells were incubated on ice at 4^°^C for 30 minutes with this rat IgG containing buffer to block any sites that may bind non-specifically the primary or secondary antibodies. 100 μL of the blocked cell suspension was transferred to each Eppendorf assay tube. The cells were then centrifuged, and the blocking buffer is aspirated. 100 μL of primary mouse monoclonal antibody (mJAA-F11) at 200 μg/ml (in excess) in assay buffer (DPBS/2%BSA) was added to the cells at the appropriate concentration and incubated in an ice bath for 60 minutes. In parallel, the same concentration of antibody was added to five Bang bead standards (Bangs Laboratories Inc., Fishers, IN, USA) that bind a known amount of mouse antibody and incubated for the same time. The cells and beads were then washed twice with wash buffer to remove unbound antibody before addition of 100 μL of the appropriate dilution of biotinylated anti-IgG secondary antibody or assay buffer (for controls). The cells and beads were incubated with the biotinylated-secondary reagent in an ice bath for 60 minutes before washing twice with wash buffer. 100 μL of streptavidin-conjugated phycoerythrin (PE) at (0.5 μg/ml) in assay buffer was added and incubated with the cells and beads on ice for 30 minutes to detect the biotinylated anti-IgG bound to JAA-F11 on the cancer cells. Cells and beads were finally washed twice with wash buffer and cells were fixed with freshly made DPBS+2% paraformaldehyde (methanol free) and 1× PBS alone was added to the beads. The beads and cell suspensions were read on a flow cytometer with the collection of a minimum of 10,000 cells. FCS data files were analyzed using TreeStar™ FlowJo software (Ashland, OR, USA) and resulting mean fluorescence intensity was used to calculate antigen binding sites per cell using the Bangs QuickCal^®^ software (Bangs Laboratories Inc., Fishers, IN, USA).

### Animals

#### Tumor measurement

Tumor sizes were measured every two to three days with surgical Vernier calipers and volumes determined by measuring the longest length (L) and the widest width (W) perpendicular to the length measurement. The equation (L × W^2^)/2, where L: is for length (mm), and W: is for width (mm) was used to determine volume.

### Tumor doubling time of selected human tumor cell lines

*In vivo* tumor doubling time was determined as described in Gordon et al (2009) using the tumor volumes acquired in the below studies [16]. Tumor doubling time (TDT) is calculated as follows: TDT = D × log (2)/ log (1 + r/100); where D is the interval in days between volume measurements, r is the rate of growth, and r/100 = (Vol 2 – Vol 1)/Vol 1 × 100%. The interval used was the difference between first day of volume measurement and last day of volume measurement before the first mouse was sacrificed.

### hJAA-F11- H2aL2a and - H3L3 immunotherapy

#### Lung cancer models

Six to eight-week-old nude mice (Jackson Laboratories, Bar Harbor, ME USA) (J: NU.007850) were housed and handled under sterile conditions. The mice received an i.p. injection of 3 mg of rabbit IgG (Sigma) (15 mg/mL), 1–3 days before humanized antibody therapy began and again at ~2 weeks intervals, to block Fc receptors. The mice were randomized into 3 groups based on maintaining equal tumor size in each group. Each group was treated with either 0.2 ml PBS, or 0.2 ml H2aL2a at 30 mg/kg, or 0.2 ml H3L3 at 30 mg/kg.

### HTB-171 human small cell lung cancer (SCLC) nude mouse xenograft model

HTB171 cells (NCI-H446) (ATCC, Manassas, VA USA) (8 × 10^6^ HTB171 tumor cells in 100 μl) were injected s.c. in the right flank of twenty-four mice. Tumors were observed after two weeks. The average tumor starting size per group was 12 mm^3^, and there was no statistical difference in the mean tumor volume of each group. Four total treatments were administered weekly via the i.p. route beginning on days 24. Tumor sizes were measured as detailed above. Mice were sacrificed, tumors were excised, and their size was re-measured. Statistical analysis of tumor size was performed as described below.

### hJAA-F11- H2aL2a and - H3L3 immunotherapy in H-520 human non-small cell lung cancer (NSCLC) nude mouse xenograft model

Mice were injected S.C. with 8 × 10^6^ cells of H520 cells (ATCC, Manassas, VA, USA) in the right flank. Tumors were observed after one week. Six total treatments with the three conditions as described above were administered weekly via i.p. route beginning on day 8. Tumor sizes were measured as described above. Mice were sacrificed on day 50, tumors were excised, and their size was re-measured. Statistical analysis of tumor size was performed as described below.

### Breast cancer models

Six to eight-week-old female SCID mice (Jackson Laboratories, Bar Harbor, ME, USA) were housed in filter bonneted microisolators with sterile bedding and autoclaved food and water ad libitum. The mice received an i.p. injection of 3 mg of rabbit IgG (Sigma) (15 mg/mL), 1–3 days before humanized antibody therapy began and again at ~2 weeks intervals, to block Fc receptors. The mice were randomized into 3 groups based on maintaining equal tumor size in each group. Each group was treated with either 0.2 ml PBS, or 0.2 ml H2aL2a at 30 mg/kg, or 0.2 ml H3L3 at 30 mg/kg.

### HCC-1806 human triple negative breast cancer SCID mouse xenograft model

On day 0, mice were injected subcutaneously (s.c.) in the right flank with 0.1 mL of 1 × 10^6^ HCC1806 human triple negative breast cancer cells in sterile 1× HBSS (Thermo Fisher Scientific Grand Island, NY, USA)/0.5% BSA. At day 14, tumor bearing mice were randomized into 3 treatment groups with the average tumor size per group was 21 mm^3^, and there was no statistical difference in the mean tumor volume of each group. The mice were injected via i.p. route with antibody or PBS on day 14 and then once a week for 3 additional weeks. The study ended and mice were sacrificed on day 38 due to ulceration at the tumor sites. Tumors were then excised, and their size was re-measured. Statistical analysis of tumor size differences was performed as described below.

### MDA-MB-231 human triple negative breast cancer SCID mouse xenograft model

On study day 0, mice were injected into the upper right abdominal mammary gland with 0.1 mL of 8 × 10^6^ MDA-MB-231 human triple negative breast cancer cells (A gift from Dr. Julian G. Cambronero, Wright University, OH, USA) in sterile 1× HBSS (Thermo Fisher Scientific Grand Island, NY, USA)/0.5% BSA. At the beginning of the study, on day 19, the average tumor size per group was 49 mm^3^, and there was no significant statistical difference in the average tumor size of each group. The mice were injected i.p. with antibody or PBS on day 19 and then once a week for 3 additional weeks. Measurement of tumor volume was as described above. Mice were sacrificed on day 47, tumors were excised, and their size was re-measured. Statistical analysis of tumor size was performed as described below.

### hJAA-F11- H2aL2a and - H3L3 immunotherapy

#### Statistical analysis of the immunocompromised models

Prism Graph PAD software (San Diego, CA, USA) was used for statistical analysis. One-or two-way ANOVA analysis with Dunnett’s test was used for determination of differences in mean tumor volume between groups, with statistical significance set at *p* < 0.05. Kaplan-Meir survival plot with Mantel-Cox Chi Square was used to determine the difference in survival between the control and experimental groups.

## SUPPLEMENTARY MATERIALS









## Abbreviations

ADCC: Antibody-Dependent Cellular Cytotoxicity
BSA: Bovine Serum Albumin
DPBS: Dulbecco’s Phosphate-Buffered Saline
ELISA: Enzyme Linked Immunosorbent Assay
Fc: Fragment Crystallizable
i.p.: Intraperitoneal
IgG: Immunoglobulin
MAHA: Mouse Antihuman Antibodies
NSCLC: Non-Small Cell Lung Cancer
OD: Optical Density
SEM: Standard Error of the Mean
s.c.: Subcutaneously
SCID: Severe Combined Immunodeficient Mice
SCLC: Small Cell Lung Cancer
SD: Standard Deviation
TF-Ag: Thomsen-Friedenreich antigen
TDT: Tumor Doubling Time
TNBC: Triple Negative Lung Cancer
