# Supplementary material for: Therapeutic efficacy of the humanized JAA-F11 anti-Thomsen-Friedenreich antibody constructs H2aL2a and H3L3 in human breast and lung cancer xenograft models
Source: Oncotarget. 2022 Oct 19;13:1155–64. doi: 10.18632/oncotarget.28282 (PMC9584441; doi:10.18632/oncotarget.28282)
Supplement: Supplementary file 1 [file oncotarget-13-28282-s001.pdf]

Therapeutic efficacy of the humanized JAA-F11 anti-Thomsen-Friedenreich antibody constructs H2aL2a and H3L3 in human breast and lung cancer xenograft models

SUPPLEMENTARY MATERIALS

Supplementary Data 1: Tumor efficacy study in mice. See Supplementary Data 1

Supplementary Data 2: Image report: expiCHOH3L3 #2 VS BSA VS FT 102617. See Supplementary Data 2

Supplementary Data 3: Image report: expicho h2al2a #2.3 vs BSA 020618. See Supplementary Data 3
