# Supplementary material for: Therapeutic efficacy of the humanized JAA-F11 anti-Thomsen-Friedenreich antibody constructs H2aL2a and H3L3 in human breast and lung cancer xenograft models
Source: Oncotarget. 2022 Oct 19;13:1155–64. doi: 10.18632/oncotarget.28282 (PMC9584441; doi:10.18632/oncotarget.28282)
Supplement: Supplementary file 3 [file oncotarget-13-28282-s003.pdf]

Supplementary Data 2: Image Report: expiCHOH3L3 #2 VS BSA VS FT 102617

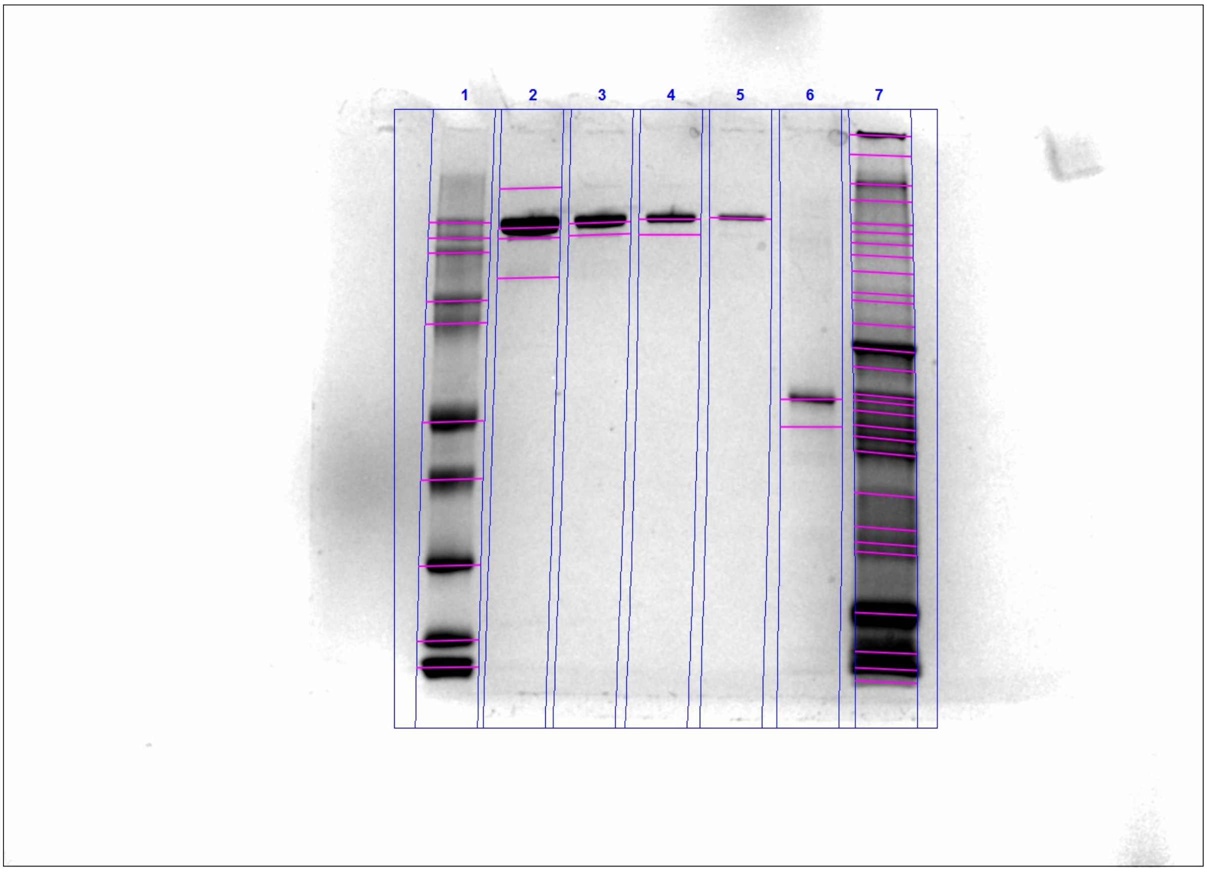

H:\expiCHOH3L3 #2 VS BSA VS FT 102617.scn

Acquisition Information

Imager	Gel Doc™ EZ
Exposure Time (sec)	1.189 (Auto - Intense Bands)
Application	Coomassie Blue
Dark Type	Referenced
Ref. Bkgd. Time (sec)	10
Flat Field	Applied
Serial Number	735BR01037
Software Version	5.2.1
Illumination Mode	White Transillumination

Image Information

Acquisition Date	10/26/2017 10:08:38 AM
User Name	Zalzala
Image Area (mm)	X: 150.0 Y: 107.8
Pixel Size (um)	X: 107.8 Y: 107.8
Data Range (Int)	830 - 3800

Analysis Settings

Detection	Lane detection:<br>Automatically detected lanes

	Band detection:<br>Automatically detected bands with custom sensitivity: 50<br>Manually adjusted bands<br><br>Lane Background Subtraction:<br>Lane background subtracted with disk size: 10<br><br>Lane width: 7.76 mm

Lane And Band Analysis

Lane 1

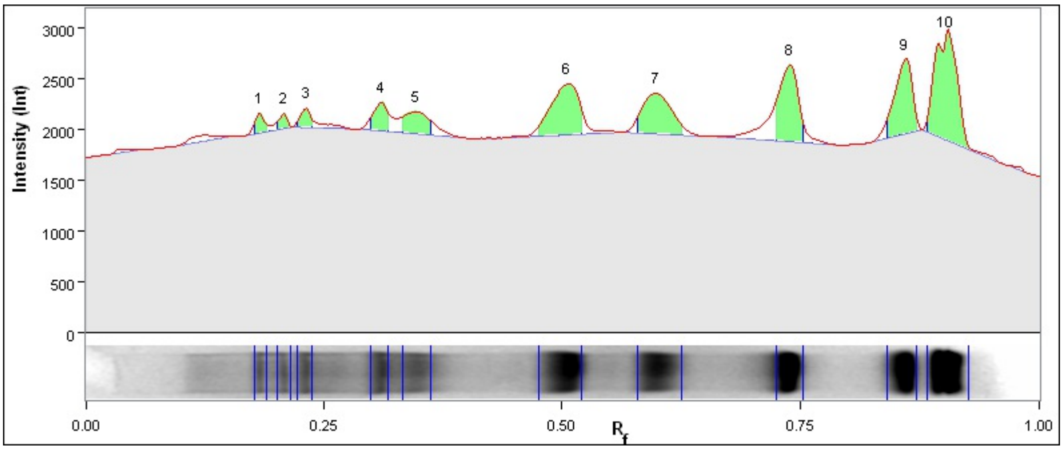

Band No.	Band Label	Mol. Wt. (KDa)	Relative Front	Volume (Int)	Abs. Quant.	Rel. Quant.	Band %	Lane %
1		N/A	0.184	107,784	N/A	N/A	1.8	1.5
2		N/A	0.209	75,384	N/A	N/A	1.3	1.1
3		N/A	0.233	124,848	N/A	N/A	2.1	1.8
4		N/A	0.311	242,352	N/A	N/A	4.2	3.4
5		N/A	0.347	316,872	N/A	N/A	5.4	4.5
6		N/A	0.506	938,880	N/A	N/A	16.1	13.2
7		N/A	0.599	748,152	N/A	N/A	12.8	10.6
8		N/A	0.738	882,864	N/A	N/A	15.1	12.5
9		N/A	0.859	835,200	N/A	N/A	14.3	11.8
10		N/A	0.903	1,561,176	N/A	N/A	26.8	22.0

Band Detection	Automatically detected bands with custom sensitivity: 50
Lane Background	Lane background subtracted with disk size: 10
Lane Width	7.76 mm

Lane 2

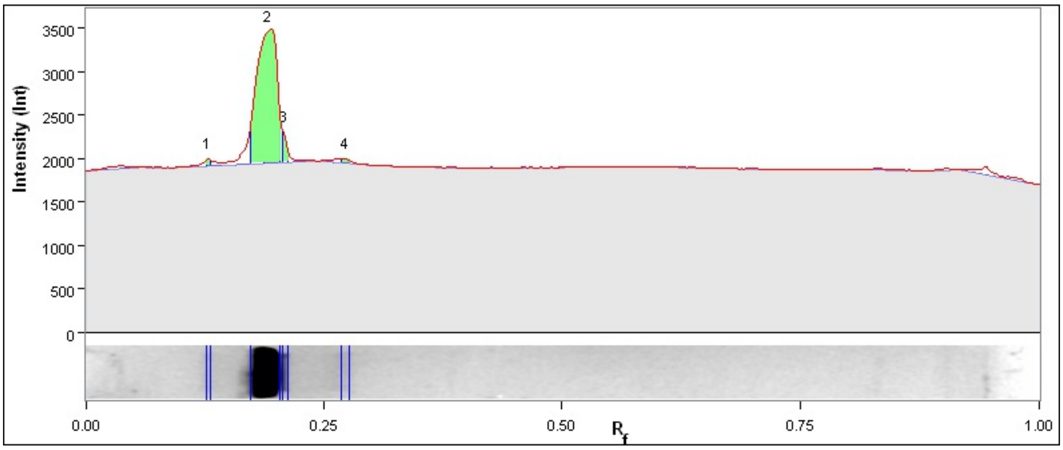

Band No.	Band Label	Mol. Wt. (KDa)	Relative Front	Volume (Int)	Abs. Quant.	Rel. Quant.	Band %	Lane %
1		N/A	0.128	21,384	N/A	N/A	1.0	0.8
2		N/A	0.192	1,961,496	N/A	N/A	94.2	72.0
3		N/A	0.209	76,824	N/A	N/A	3.7	2.8
4		N/A	0.273	21,600	N/A	N/A	1.0	0.8

Band Detection	Automatically detected bands with custom sensitivity: 50
Lane Background	Lane background subtracted with disk size: 10
Lane Width	7.76 mm

Lane 3

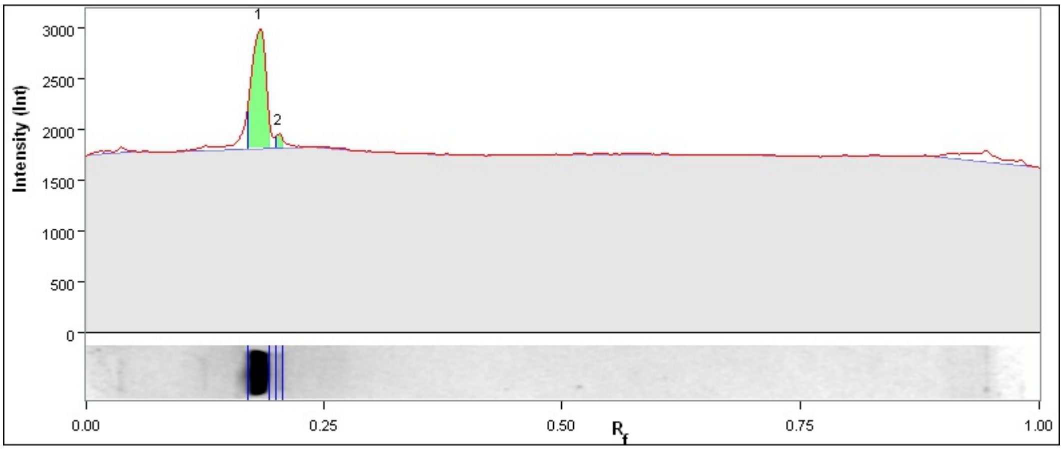

Band No.	Band Label	Mol. Wt. (KDa)	Relative Front	Volume (Int)	Abs. Quant.	Rel. Quant.	Band %	Lane %
1		N/A	0.184	1,083,744	N/A	N/A	95.3	55.7
2		N/A	0.203	53,928	N/A	N/A	4.7	2.8

Band Detection	Automatically detected bands with custom sensitivity: 50
Lane Background	Lane background subtracted with disk size: 10
Lane Width	7.76 mm

Lane 4

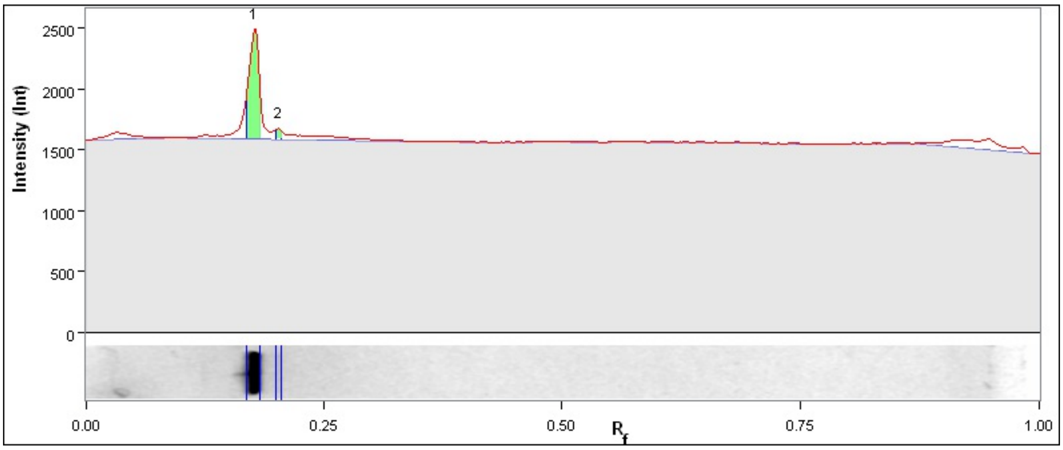

Band No.	Band Label	Mol. Wt. (KDa)	Relative Front	Volume (Int)	Abs. Quant.	Rel. Quant.	Band %	Lane %
1		N/A	0.178	619,200	N/A	N/A	95.0	37.2
2		N/A	0.203	32,832	N/A	N/A	5.0	2.0

Band Detection	Automatically detected bands with custom sensitivity: 50
Lane Background	Lane background subtracted with disk size: 10
Lane Width	7.76 mm

Lane 5

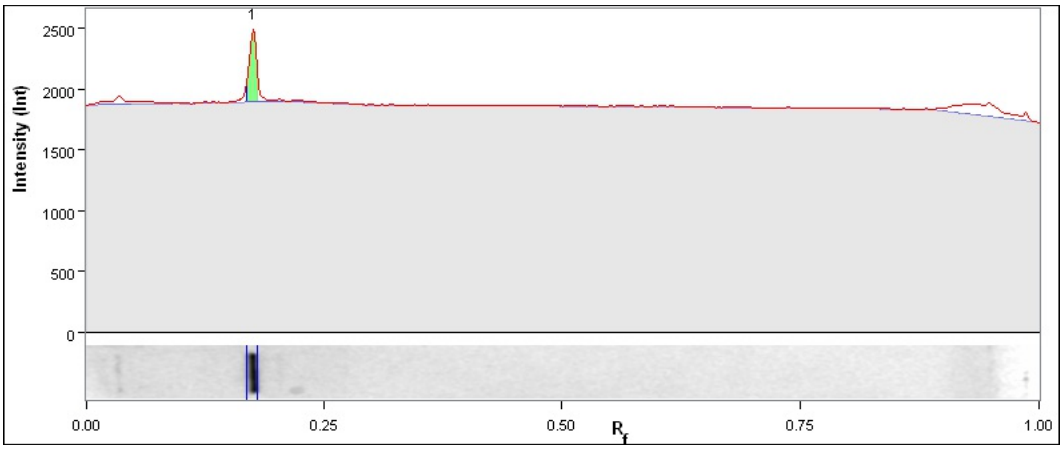

Band No.	Band Label	Mol. Wt. (KDa)	Relative Front	Volume (Int)	Abs. Quant.	Rel. Quant.	Band %	Lane %
1		N/A	0.177	257,976	N/A	N/A	100.0	30.7

Band Detection	Automatically detected bands with custom sensitivity: 50
Lane Background	Lane background subtracted with disk size: 10
Lane Width	7.76 mm

Lane 6

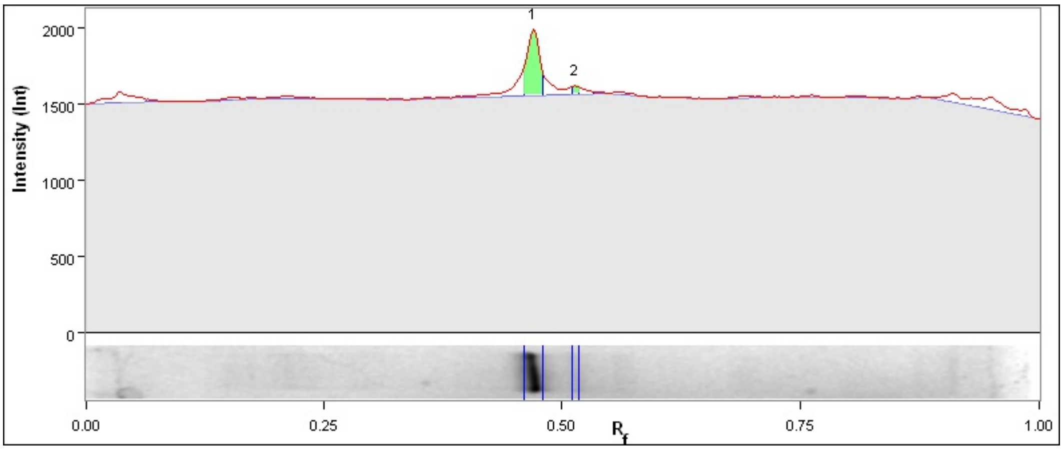

Band No.	Band Label	Mol. Wt. (KDa)	Relative Front	Volume (Int)	Abs. Quant.	Rel. Quant.	Band %	Lane %
1		N/A	0.469	409,608	N/A	N/A	93.6	29.1
2		N/A	0.514	28,152	N/A	N/A	6.4	2.0

Band Detection	Automatically detected bands with custom sensitivity: 50
Lane Background	Lane background subtracted with disk size: 10
Lane Width	7.76 mm

Lane 7

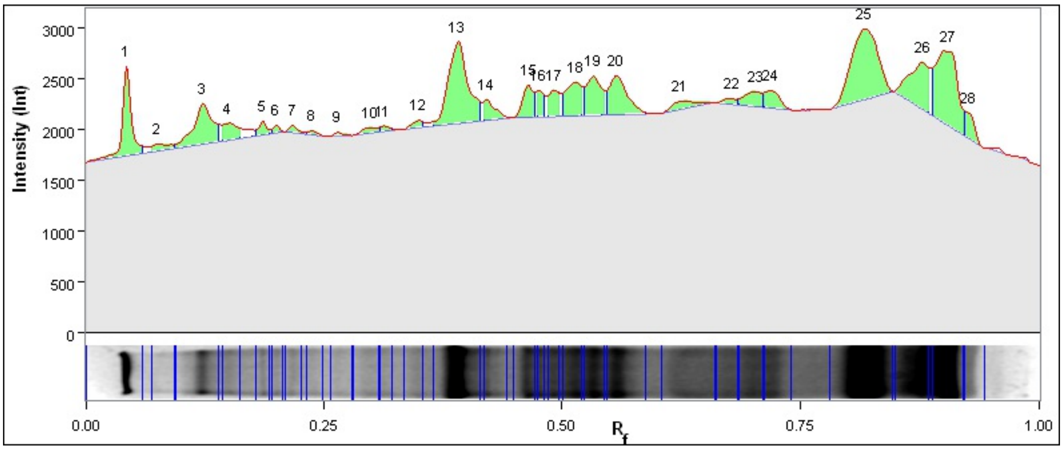

Band No.	Band Label	Mol. Wt. (KDa)	Relative Front	Volume (Int)	Abs. Quant.	Rel. Quant.	Band %	Lane %
1		N/A	0.043	528,552	N/A	N/A	6.4	6.1
2		N/A	0.075	76,896	N/A	N/A	0.9	0.9
3		N/A	0.123	511,488	N/A	N/A	6.2	5.9
4		N/A	0.149	168,552	N/A	N/A	2.1	2.0
5		N/A	0.187	73,368	N/A	N/A	0.9	0.8
6		N/A	0.201	33,264	N/A	N/A	0.4	0.4
7		N/A	0.219	38,808	N/A	N/A	0.5	0.4
8		N/A	0.238	23,544	N/A	N/A	0.3	0.3
9		N/A	0.265	19,800	N/A	N/A	0.2	0.2
10		N/A	0.299	54,360	N/A	N/A	0.7	0.6
11		N/A	0.312	31,536	N/A	N/A	0.4	0.4
12		N/A	0.350	49,248	N/A	N/A	0.6	0.6
13		N/A	0.390	1,069,344	N/A	N/A	13.0	12.4
14		N/A	0.421	150,984	N/A	N/A	1.8	1.7
15		N/A	0.465	239,256	N/A	N/A	2.9	2.8
16		N/A	0.475	113,472	N/A	N/A	1.4	1.3
17		N/A	0.492	187,128	N/A	N/A	2.3	2.2
18		N/A	0.515	338,112	N/A	N/A	4.1	3.9
19		N/A	0.533	397,872	N/A	N/A	4.8	4.6
20		N/A	0.557	473,688	N/A	N/A	5.8	5.5
21		N/A	0.624	125,640	N/A	N/A	1.5	1.5
22		N/A	0.678	52,200	N/A	N/A	0.6	0.6
23		N/A	0.703	179,568	N/A	N/A	2.2	2.1
24		N/A	0.719	172,872	N/A	N/A	2.1	2.0
25		N/A	0.816	1,291,104	N/A	N/A	15.7	15.0
26		N/A	0.879	572,400	N/A	N/A	7.0	6.6
27		N/A	0.905	1,074,456	N/A	N/A	13.1	12.4
28		N/A	0.926	172,080	N/A	N/A	2.1	2.0

Band Detection	Automatically detected bands with custom sensitivity: 50
Lane Background	Lane background subtracted with disk size: 10
Lane Width	7.76 mm
