# Supplementary material for: Therapeutic efficacy of the humanized JAA-F11 anti-Thomsen-Friedenreich antibody constructs H2aL2a and H3L3 in human breast and lung cancer xenograft models
Source: Oncotarget. 2022 Oct 19;13:1155–64. doi: 10.18632/oncotarget.28282 (PMC9584441; doi:10.18632/oncotarget.28282)
Supplement: Supplementary file 4 [file oncotarget-13-28282-s004.pdf]

Supplementary Data 3: Image Report: expicho h2al2a #2.3 vs BSA 020618

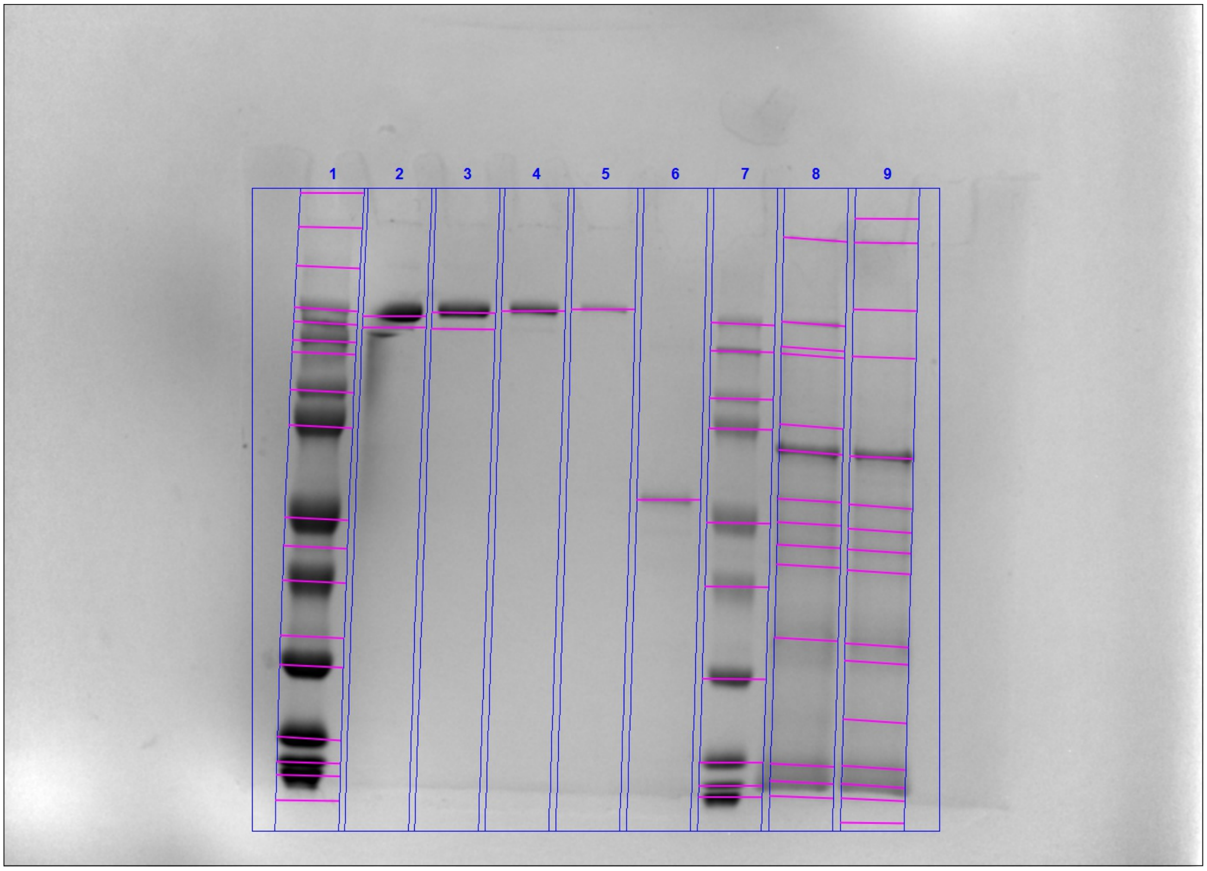

H:\expicho h2al2a #2.3 vs BSA 020618.scn

Acquisition Information

Imager	Gel Doc™ EZ
Exposure Time (sec)	1.226 (Auto - Intense Bands)
Application	Coomassie Blue
Dark Type	Referenced
Ref. Bkgd. Time (sec)	10
Flat Field	Applied
Serial Number	735BR01037
Software Version	5.2.1
Illumination Mode	White Transillumination

Image Information

Acquisition Date	2/8/2018 4:23:54 PM
User Name	Zalzala
Image Area (mm)	X: 150.0 Y: 107.8
Pixel Size (um)	X: 107.8 Y: 107.8
Data Range (Int)	724 - 3804

Analysis Settings

Detection	Lane detection:<br>Automatically detected lanes with manual adjustments<br><br>Band detection:<br>Automatically detected bands with custom sensitivity: 50<br>Manually adjusted bands<br><br>Lane Background Subtraction:<br>Lane background subtracted with disk size: 10<br><br>Lane width: 7.97 mm

Lane And Band Analysis

Lane 1

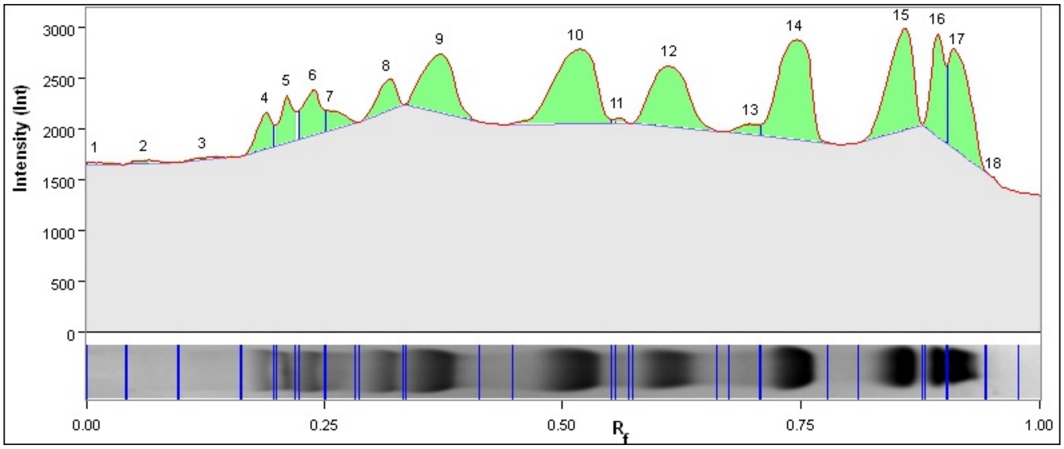

Band No.	Band Label	Mol. Wt. (KDa)	Relative Front	Volume (Int)	Abs. Quant.	Rel. Quant.	Band %	Lane %
1		N/A	0.008	28,342	N/A	N/A	0.2	0.2
2		N/A	0.062	57,868	N/A	N/A	0.4	0.4
3		N/A	0.123	46,324	N/A	N/A	0.3	0.3
4		N/A	0.189	391,608	N/A	N/A	2.7	2.7
5		N/A	0.210	444,740	N/A	N/A	3.1	3.1
6		N/A	0.239	595,774	N/A	N/A	4.1	4.1
7		N/A	0.257	245,236	N/A	N/A	1.7	1.7
8		N/A	0.316	432,752	N/A	N/A	3.0	3.0
9		N/A	0.371	1,336,588	N/A	N/A	9.3	9.2
10		N/A	0.515	2,274,316	N/A	N/A	15.8	15.7
11		N/A	0.559	33,152	N/A	N/A	0.2	0.2
12		N/A	0.613	1,634,290	N/A	N/A	11.3	11.3
13		N/A	0.698	149,850	N/A	N/A	1.0	1.0
14		N/A	0.744	2,349,278	N/A	N/A	16.3	16.2
15		N/A	0.857	1,832,980	N/A	N/A	12.7	12.6
16		N/A	0.894	1,003,070	N/A	N/A	7.0	6.9
17		N/A	0.914	1,569,614	N/A	N/A	10.9	10.8
18		N/A	0.953	5,994	N/A	N/A	0.0	0.0

Band Detection	Automatically detected bands with custom sensitivity: 50
Lane Background	Lane background subtracted with disk size: 10
Lane Width	7.97 mm

Lane 2

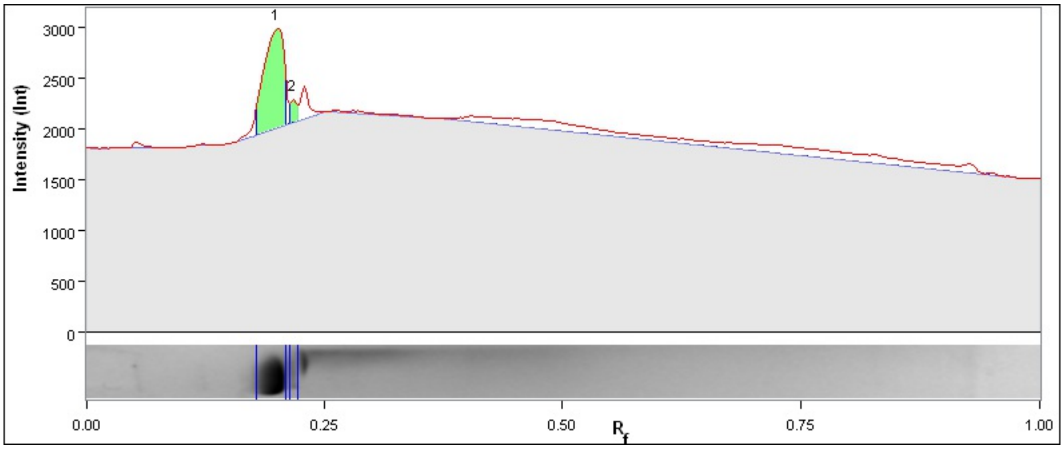

Band No.	Band Label	Mol. Wt. (KDa)	Relative Front	Volume (Int)	Abs. Quant.	Rel. Quant.	Band %	Lane %
1		N/A	0.200	1,270,876	N/A	N/A	92.7	33.9
2		N/A	0.217	100,344	N/A	N/A	7.3	2.7

Band Detection	Automatically detected bands with custom sensitivity: 50
Lane Background	Lane background subtracted with disk size: 10
Lane Width	7.97 mm

Lane 3

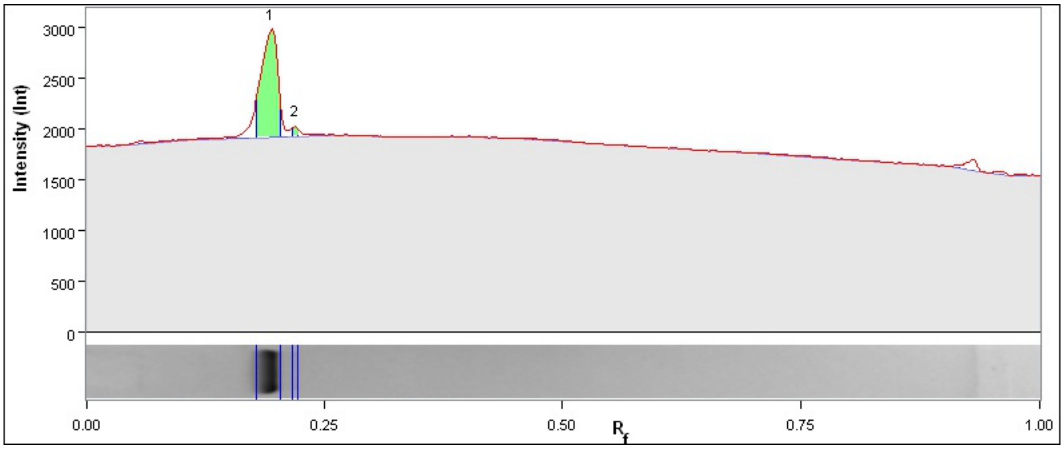

Band No.	Band Label	Mol. Wt. (KDa)	Relative Front	Volume (Int)	Abs. Quant.	Rel. Quant.	Band %	Lane %
1		N/A	0.194	1,082,250	N/A	N/A	97.2	61.8
2		N/A	0.220	31,598	N/A	N/A	2.8	1.8

Band Detection	Automatically detected bands with custom sensitivity: 50
Lane Background	Lane background subtracted with disk size: 10
Lane Width	7.97 mm

Lane 4

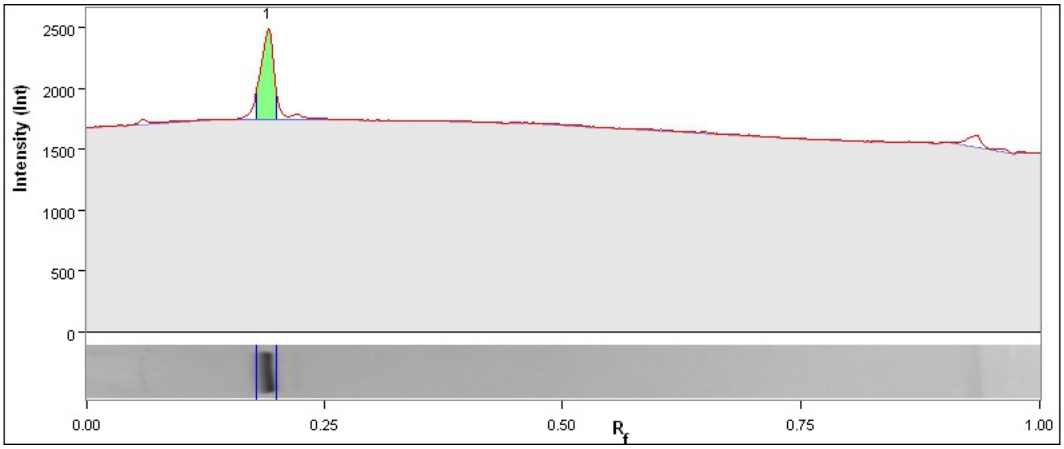

Band No.	Band Label	Mol. Wt. (KDa)	Relative Front	Volume (Int)	Abs. Quant.	Rel. Quant.	Band %	Lane %
1		N/A	0.192	640,692	N/A	N/A	100.0	52.3

Band Detection	Automatically detected bands with custom sensitivity: 50
Lane Background	Lane background subtracted with disk size: 10
Lane Width	7.97 mm

Lane 5

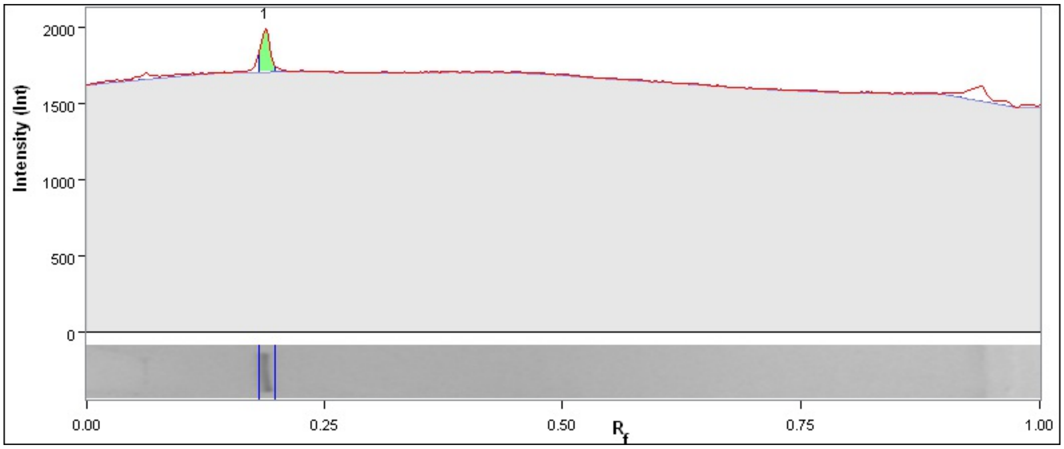

Band No.	Band Label	Mol. Wt. (KDa)	Relative Front	Volume (Int)	Abs. Quant.	Rel. Quant.	Band %	Lane %
1		N/A	0.189	177,822	N/A	N/A	100.0	25.7

Band Detection	Automatically detected bands with custom sensitivity: 50
Lane Background	Lane background subtracted with disk size: 10
Lane Width	7.97 mm

Lane 6

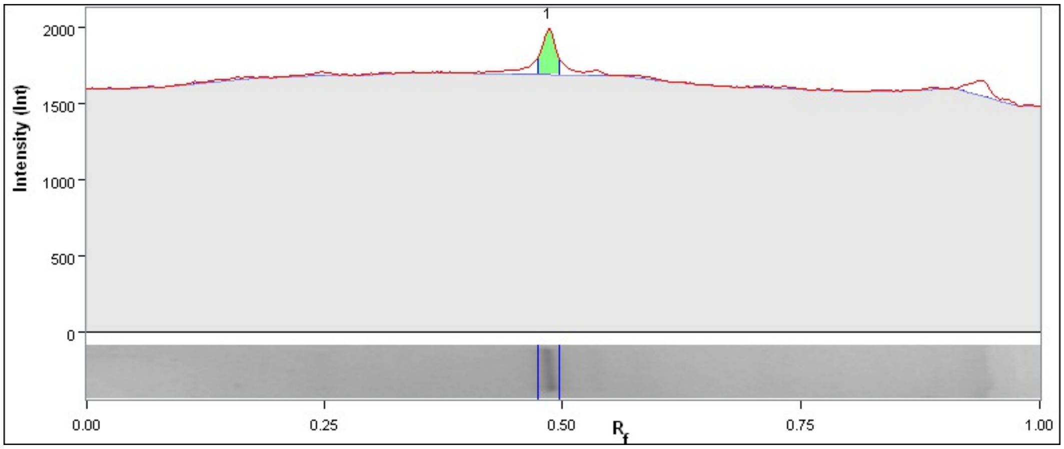

Band No.	Band Label	Mol. Wt. (KDa)	Relative Front	Volume (Int)	Abs. Quant.	Rel. Quant.	Band %	Lane %
1		N/A	0.485	294,150	N/A	N/A	100.0	30.2

Band Detection	Automatically detected bands with custom sensitivity: 50
Lane Background	Lane background subtracted with disk size: 10
Lane Width	7.97 mm

Lane 7

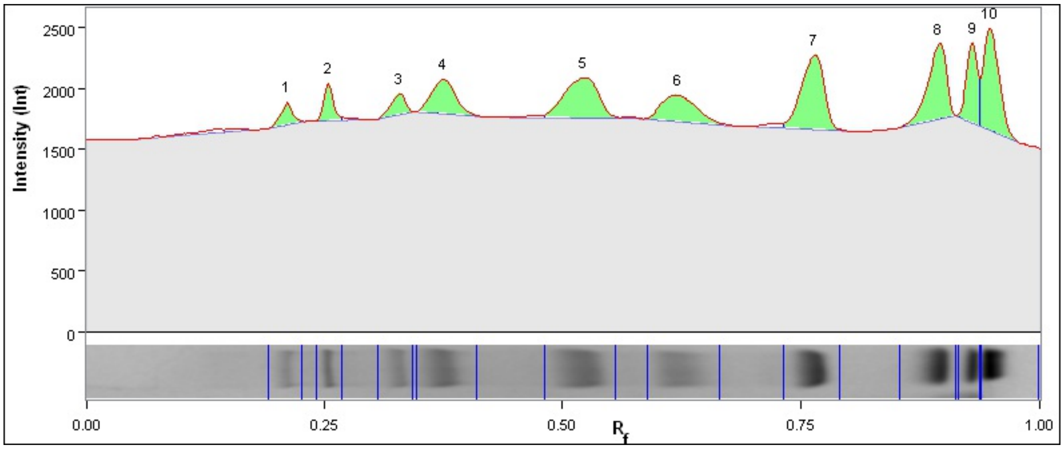

Band No.	Band Label	Mol. Wt. (KDa)	Relative Front	Volume (Int)	Abs. Quant.	Rel. Quant.	Band %	Lane %
1		N/A	0.212	177,082	N/A	N/A	2.9	2.7
2		N/A	0.255	216,450	N/A	N/A	3.5	3.4
3		N/A	0.328	191,290	N/A	N/A	3.1	3.0
4		N/A	0.375	535,464	N/A	N/A	8.7	8.3
5		N/A	0.521	882,154	N/A	N/A	14.3	13.7
6		N/A	0.621	594,294	N/A	N/A	9.6	9.2
7		N/A	0.764	1,087,948	N/A	N/A	17.6	16.9
8		N/A	0.894	903,762	N/A	N/A	14.6	14.0
9		N/A	0.930	568,764	N/A	N/A	9.2	8.8
10		N/A	0.948	1,032,078	N/A	N/A	16.7	16.0

Band Detection	Automatically detected bands with custom sensitivity: 50
Lane Background	Lane background subtracted with disk size: 10
Lane Width	7.97 mm

Lane 8

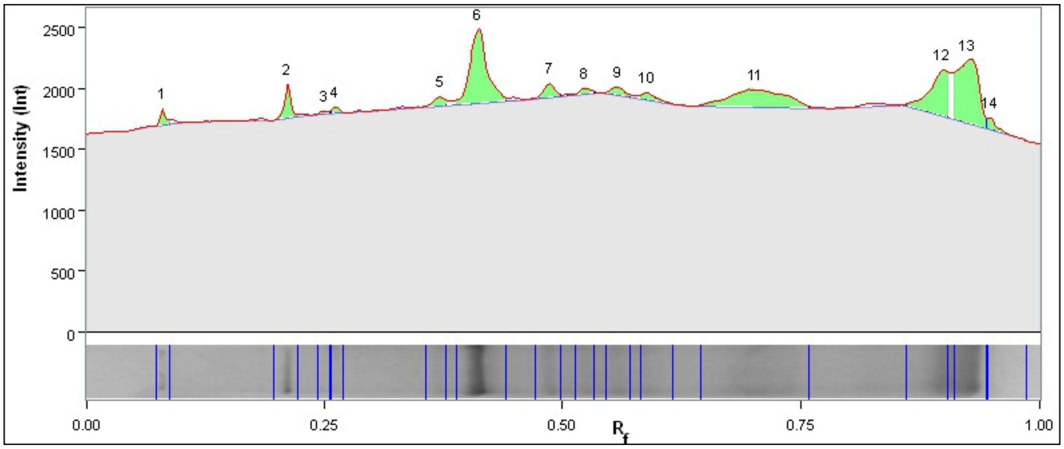

Band No.	Band Label	Mol. Wt. (KDa)	Relative Front	Volume (Int)	Abs. Quant.	Rel. Quant.	Band %	Lane %
1		N/A	0.080	62,456	N/A	N/A	1.8	1.7
2		N/A	0.212	149,924	N/A	N/A	4.4	4.0
3		N/A	0.251	16,280	N/A	N/A	0.5	0.4
4		N/A	0.261	26,492	N/A	N/A	0.8	0.7
5		N/A	0.371	66,378	N/A	N/A	1.9	1.8
6		N/A	0.412	785,658	N/A	N/A	23.1	20.9
7		N/A	0.487	95,756	N/A	N/A	2.8	2.5
8		N/A	0.523	43,660	N/A	N/A	1.3	1.2
9		N/A	0.558	70,522	N/A	N/A	2.1	1.9
10		N/A	0.588	67,118	N/A	N/A	2.0	1.8
11		N/A	0.702	617,160	N/A	N/A	18.1	16.4
12		N/A	0.898	496,022	N/A	N/A	14.6	13.2
13		N/A	0.925	841,380	N/A	N/A	24.7	22.3
14		N/A	0.948	69,116	N/A	N/A	2.0	1.8

Band Detection	Automatically detected bands with custom sensitivity: 50
Lane Background	Lane background subtracted with disk size: 10
Lane Width	7.97 mm

Lane 9

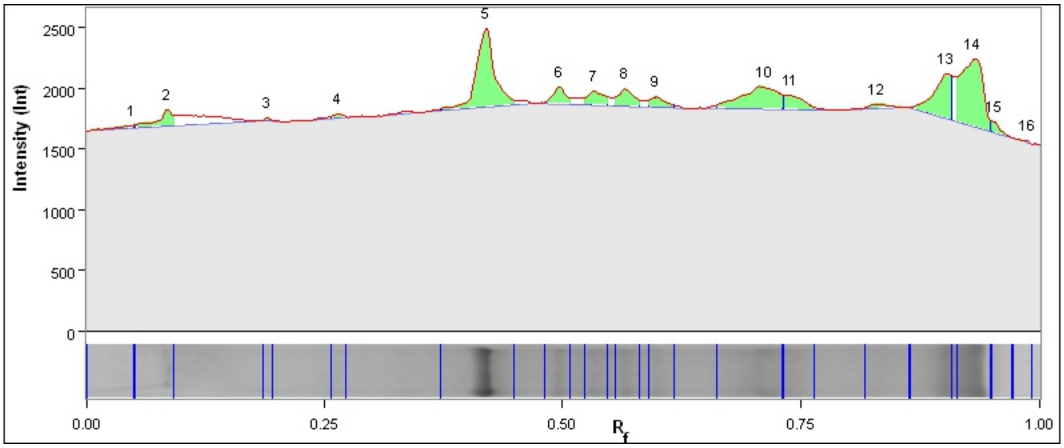

Band No.	Band Label	Mol. Wt. (KDa)	Relative Front	Volume (Int)	Abs. Quant.	Rel. Quant.	Band %	Lane %

1		N/A	0.048	32,782	N/A	N/A	0.9	0.8
2		N/A	0.086	152,736	N/A	N/A	4.1	3.6
3		N/A	0.190	11,174	N/A	N/A	0.3	0.3
4		N/A	0.264	23,902	N/A	N/A	0.6	0.6
5		N/A	0.420	844,636	N/A	N/A	22.7	19.7
6		N/A	0.496	133,496	N/A	N/A	3.6	3.1
7		N/A	0.534	138,380	N/A	N/A	3.7	3.2
8		N/A	0.566	159,840	N/A	N/A	4.3	3.7
9		N/A	0.598	95,756	N/A	N/A	2.6	2.2
10		N/A	0.712	492,100	N/A	N/A	13.2	11.5
11		N/A	0.739	164,354	N/A	N/A	4.4	3.8
12		N/A	0.830	52,984	N/A	N/A	1.4	1.2
13		N/A	0.902	466,274	N/A	N/A	12.5	10.9
14		N/A	0.930	883,930	N/A	N/A	23.8	20.6
15		N/A	0.952	62,604	N/A	N/A	1.7	1.5
16		N/A	0.988	5,550	N/A	N/A	0.1	0.1

Band Detection	Automatically detected bands with custom sensitivity: 50
Lane Background	Lane background subtracted with disk size: 10
Lane Width	7.97 mm
